# Association between shared medical appointments and weight loss outcomes and anti‐obesity medication use in patients with obesity

**DOI:** 10.1002/osp4.406

**Published:** 2020-02-25

**Authors:** Kelly Shibuya, Xinge Ji, Elizabeth R. Pfoh, Alex Milinovich, Wayne Weng, Janine Bauman, Rahul Ganguly, Anita D. Misra‐Hebert, Todd M. Hobbs, Michael W. Kattan, Kevin M. Pantalone, Abhilasha Ramasamy, Bartolome Burguera

**Affiliations:** ^1^ Cleveland Clinic Lerner College of Medicine Case Western Reserve University Cleveland Ohio; ^2^ Quantitative Health Sciences Cleveland Clinic Cleveland Ohio; ^3^ Department of Internal Medicine Cleveland Clinic Community Care Cleveland Ohio; ^4^ Health Economics and Outcomes Research Novo Nordisk Inc. Plainsboro New Jersey; ^5^ Diabetes, Chief Medical Officer Novo Nordisk Inc. Plainsboro New Jersey; ^6^ Endocrinology and Metabolism Institute Cleveland Clinic Cleveland New Jersey; ^7^ National Diabetes and Obesity Research Institute Tradition Mississippi

**Keywords:** Obesity, weight management, shared medical appointments

## Abstract

**Objective:**

In shared medical appointments (SMAs), multiple patients with a similar clinical diagnosis are seen by a multidisciplinary team for interactive group sessions. Very few studies have specifically studied SMAs and weight loss in patients with obesity. This study compared weight loss outcomes and anti‐obesity medication (AOM) access between patients with obesity managed through (SMAs) versus individual appointments.

**Methods:**

Retrospective study of adults seen for obesity between September 2014 and February 2017 at Cleveland Clinic Institute of Endocrinology and Metabolism. Percent weight loss from baseline was compared between two propensity score‐matched populations: patients who attended ≥1 SMA and patients managed with individual medical appointments.

**Results:**

From all eligible patients identified (n=310 SMA, n=1,993 non‐SMA), 301 matched pairs were evaluated for weight loss. The SMA group (n=301) lost a mean of 4.2%, 5.2% and 3.8% of baseline weight over 6, 12 and 24 months; the non‐SMA group (n=301) lost significantly less weight (1.5%, 1.8% and 1.6%, respectively) (paired *t*‐test, *P*<.05). All patients were eligible for US Food and Drug Administration‐approved AOMs based on obesity diagnosis; however, 49.8% (150/301) of matched SMA patients were prescribed an AOM versus 12.3% (37/301) of matched non‐SMA patients.

**Conclusion:**

This study suggests that SMAs may offer a promising alterative for obesity management and one that may facilitate greater utilization of AOMs. In propensity score‐matched cohorts, SMAs were associated with greater weight loss outcomes when compared to usual care facilitated through individual medical appointments alone.

## 1 INTRODUCTION

The recognition of obesity as a chronic disease by the American Medical Association in 2013^1^ encourages physicians to be on the forefront of not only treating obesity‐related comorbidities but also the primary problem of weight management. However, due to limitations in time and resources, physicians often focus on the consequences of obesity, rather than the primary problem of obesity itself. Patients may be prescribed antihypertensives or diabetes medications, but typically only receive brief advice on weight management.^2^ Additionally, anti‐obesity medications (AOMs) are prescribed to <2% of patients who are eligible.^3^ These current methods of obesity treatment do not provide patients with the intensive interventions needed for long‐term weight loss success.^4^

Shared medical appointments (SMAs) may be one method to deliver effective and efficient obesity treatment.^5^ SMAs are group medical visits where multiple patients with a similar clinical diagnosis are seen by a multidisciplinary team for interactive group sessions that typically last 60 to 90 min each.^6^ Theorized beneficial aspects of SMAs include fostering patient‐patient and patient‐caregiver relationships, co‐learning from others with the same condition, gaining inspiration from others' successes, better understanding of patients' needs by providers, greater provider‐patient interaction time and improved patient trust in providers.^7^ Patients experience the support of other patients and often learn more than during a traditional appointment as a result of the longer sessions and from questions and comments shared by other SMA participants.^8^ Data on the use of SMAs continue to accumulate, particularly in the field of diabetes.^9^, ^10^, ^11^, ^12^, ^13^, ^14^, ^15^, ^16^ Reviews of such research have concluded that while there are indications of improved outcomes in patients managed with SMAs, the small size and heterogeneity of studies thus far does not allow for conclusions to be drawn from the collective body of research.^16^, ^17^

Very few studies have specifically studied SMAs as a weight loss strategy in patients with obesity.^18^, ^19^, ^20^ This study examines the hypothesis that patients involved in weight loss‐focused SMAs would experience greater weight loss over a 2‐year period compared to patients who received standard obesity management through individual medical appointments alone.

## 2 METHODS

### 2.1 Study design and population

This retrospective study included patients age ≥18 years with body mass index (BMI) ≥27 kg m^−2^ who were seen in the Cleveland Clinic Department of Endocrinology with an International Classification of Diseases (ICD) encounter diagnoses for obesity or morbid obesity (ICD‐9: 278.00, 278.01, V85.41, V85.43; ICD10: E66.01, E66.09, E66.9, Z68.41, Z68.43) between September 2014 and February 2017. Patients in the SMA group attended at least 1 SMA during this study period with the option of scheduling supplemental individual medical appointments, as needed. Patients in the non‐SMA group were those who had an ICD‐9 encounter diagnosis code for obesity or morbid obesity during an individual medical appointment with an endocrinologist during the same study period. Data were collected from extraction of electronic medical record (EMR) data. This study was approved by the Institutional Review Board at the Cleveland Clinic.

### 2.2 Obesity shared medical appointments

**Figure 1:**
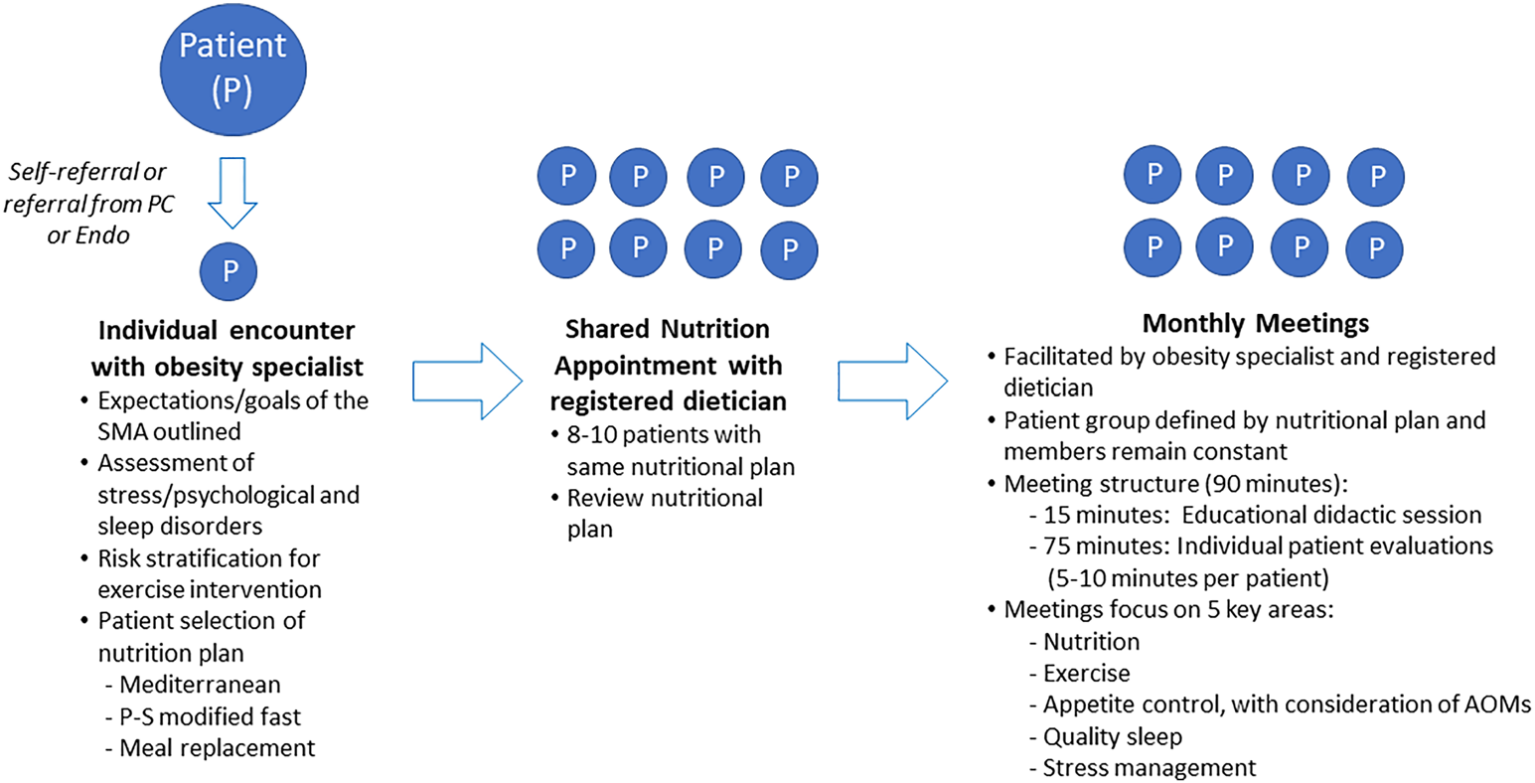
Shared medical appointments (SMAs) at the Cleveland Clinic Department of Endocrinology‐Process and structure. AOMs: anti‐obesity medications; Endo: endocrinologist; PC: primary care; P‐S: protein‐sparing; SMA: shared medical appointment [Colour figure can be viewed at wileyonlinelibrary.com]

The process and structure of SMAs provided through the Cleveland Clinic Department of Endocrinology are illustrated in Figure 1. Patients with obesity were referred for an obesity SMA through self‐referral or referral from their primary care provider or endocrinologist. Patients who present with the primary intent of weight loss are generally good candidates for SMAs. In general, referral suitability was a judgment call on the part of the clinician based on a sense of whether the patient would benefit from the socialization aspect and/or accountability structure of SMAs. Once a patient was referred, the first step was an individual encounter with an obesity specialist (physician or nurse practitioner). This initial meeting entailed an overview of the expectations and goals of the SMAs and evaluation of the patient for possible stress/psychological disorders, sleep disorders and risk stratification to help guide an exercise intervention strategy. At this visit, patients selected a nutrition plan from one of three options: Mediterranean, protein‐sparing modified fast or meal replacement. The meal replacement plan was based on a recommendation of 500 to 700 calories per meal; meal replacements could be bars/shakes/snacks meeting the following specifications: 200 to 300 calories, no more than 30 g of carbohydrates and 10 to 30 g of protein. Patients were provided examples of commercially available meal replacement products (bars and shakes) that fit the criteria as part of their educational materials, as well as recipes for bars and shakes for patients who preferred to make their own.

Based on their nutrition plan selection, patients attended a shared nutrition appointment in a group of 8 to 10 patients, led by a registered dietician, to review the nutrition plan. Patients then met monthly in 90‐min SMAs in groups of 8 to 10 patients. These monthly meetings were facilitated by an obesity specialist (primary care provider with primary interest in obesity, board certified obesity medicine provider, or endocrinologist) and registered dietician. The SMA groups were defined by the chosen nutrition plan, and group members remained constant throughout the program. The initial 15 min of each monthly SMA visit was spent in an educational didactic session, and the remainder of the time was spent performing individual patient evaluations. During the individual evaluations, each patient spent 5‐ to 10‐min consulting with the providers, like a traditional individual medical appointment. During this time, others in the group observed and learned from shared experiences of their peers. The SMAs focused on five areas that are key to obesity treatment: nutrition, exercise, appetite control with consideration of AOMs, sleep quality and stress management.

### 2.3 Usual care individual medical appointments

This reference group included all patients with an ICD‐9 encounter diagnosis of obesity or morbid obesity during the study period who had been seen in the Department of Endocrinology by a general practicing endocrinologist who did not conduct SMAs. While it is presumed that obesity was addressed in the appointment, it may not have been the primary focus. The intensity and frequency of obesity treatment provided to these patients were at the discretion of the provider.

### 2.4 Measures

The primary outcome of this study was percent weight loss from baseline weight. Baseline weight was defined as the recorded weight at the enrolment of the SMA program for the SMA group or the recorded weight at the first individual appointment during the study period for the non‐SMA group. Outcome weight was defined by the weight measured at the nearest outpatient visit, as recorded in the EMR, at 6 months (±30 d), 1 year (±30 d) and 2 years (±60 d). Other variables collected from the EMR included age at enrollment, gender, race, marital status, insurance type, median household income (defined as the 2011‐2015 5‐year estimates of median household income at the block group level obtained from the American Community Survey conducted by the U.S. Census Bureau [21]), weight, blood pressure, selected comorbidities using ICD‐9 and ‐10 codes (type 2 diabetes (T2D), hypertension, dyslipidemia) and use of an AOM (defined as prescription of at least one AOM, phentermine‐hydrochloride, phentermine‐topiramate (Qsymia), lorcaserin (Belviq), bupropion‐naltrexone (Contrave), liraglutide (Saxenda, Saxenda XR) or orlistat (Xenical, Alli), for at least 90 d within the study period), number of SMA visits and duration of SMA enrolment (time between first and last SMA visits).

### 2.5 Statistical analysis

Descriptive and outcome data were analyzed using chi‐square for categorical variables and *t* test for continuous variables for unmatched comparisons. Prior to propensity matching, weight loss was compared between SMA and non‐SMA groups while stratified by use of an AOM. To account for differences in baseline characteristics, a logistic regression model including age, gender, race, marital status, insurance type, median household income, baseline weight (kg) and diagnosis of T2D at baseline was used to generate a propensity score for attending an SMA. Patients in the SMA group were matched 1:1 with patients who did not attend an SMA (non‐SMA group) using a caliper width of 0.2. Patients with missing weight values were excluded from that specific analysis. Post‐match analyses were performed using paired *t* test. Statistical analysis was performed using R statistical software.

## 3 RESULTS

### 3.1 Population description prior to propensity matching

A total of 310 patients were identified in the SMA group and 1,993 patients in the (non‐SMA) group. In the SMA group, the median number of SMAs was 4 (IQR 2‐8; range, 2‐37) and the median duration of enrolment (SMA participation) was 1.2 years (IQR 0.5, 2.0; maximum 3.6 years). Baseline characteristics are shown in Table 1. Patients in the SMA group were more likely to be female (83.9% vs. 68.2%), participate in the Cleveland Clinic Employee Health Plan (21.2% vs. 5.4%), had a higher initial BMI (40.6 kg m^−2^ vs. 38.6 kg m^−2^), had a lower income ($50,715 vs. $55,110) and had a lower baseline prevalence of T2D (37.4% vs. 53.2%) compared to those in the non‐SMA group (all *P*<.001). AOMs were prescribed for 50.3% (n=156) of patients in the SMA group and 7.1% (n=142) of patients in the control group (Table 2). The most used AOM was phentermine hydrochloride, an AOM approved for short‐term use (46.5% of SMA patients; 4.7% of non‐SMA patients). Patients in the SMA group had a mean (SD) of 6.19 (3.13) endocrinology visits as compared to a mean of 2.14 (2.07) visits in the non‐SMA group.

**Table 1:** Baseline demographics of total population

Variable Median (IQR) or n (%)	SMAN=310	Non‐SMA N=1,993	*P*‐value
Age (years)	52.34 (42.16, 60.52)	51.44 (38.86, 60.64)	.26
Female gender	260 (83.9%)	1,359 (68.2%)	<.001
Race			<.001
White	157 (50.6%)	1,459 (73.2%)	
Black	141 (45.5%)	361 (18.1%)	
Other	12 (3.9%)	173 (8.7%)	
Marital status			.01
Married	143 (46.1%)	1,090 (54.7%)	
Other	167 (53.9%)	903 (45.3%)	
Insurance			<.001
EHP	63 (21.2%)	99 (5.4%)	
Medicaid	50 (16.8%)	177 (9.7%)	
Medicare	74 (24.9%)	517 (28.5%)	
Other	2 (0.7%)	243 (13.4%)	
Private health insurance	108 (36.4%)	781 (43%)	
Median income (US Dollars)	50,715 (32,357, 63,445)	55,110 (44,477, 70,463)	<.001
BMI, kg m^−2^	40.59 (35.84, 45.94)	38.56 (33.92, 44)	<.001
Weight, kg	111.24 (95.5, 131.39)	109.32 (94.8, 128.82)	.08
Blood pressure, mmHg			
Systolic	130 (120, 140)	130 (120, 140)	.40
Diastolic	78 (68, 84)	78 (70, 84)	.98
Comorbidities			
T2D	116 (37.4%)	1,060 (53.2%)	<.001
Hypertension	203 (65.5%)	1,235 (62%)	<.01
Dyslipidemia	187 (60.3%)	1,218 (61.1%)	<.001

*Note.* Continuous variables summarized as median, IQR (interquartile range) and categorical variables summarized as N (%).

Abbreviations: BMI: body mass index; EHP: Cleveland Clinic Employee Health Plan; IQR: interquartile range; SMA: shared medical appointments; T2D: type 2 diabetes mellitus.

**Table 2:** AOM prescription rates in the overall study population and propensity‐matched cohorts

Overall Study Population		
	SMA(N=310) n (%)	Control (N=1,993) n (%)
At least one AOM	156 (50.3)	142 (7.1)
Phentermine hydrochloride	144 (46.5)	93 (4.7)
Phentermine‐topiramate	27 (8.7)	18 (0.9)
Naltrexone‐bupropion	38 (12.3)	53 (2.7)
Lorcaserin	1 (0.3)	7 (0.4)
Liraglutide	0	2 (0.1)
Orlistat	3 (1.0)	3 (0.2)
**Propensity Score‐Matched Cohorts**		
	**SMA(N=310) n (%)**	**Non‐SMA(N=301) n (%)**
At least one AOM	150 (49.8)	37 (12.3)
Phentermine hydrochloride	138 (45.8)	26 (8.6)
Phentermine‐topiramate	26 (8.6)	2 (0.7)
Naltrexone‐bupropion	36 (12.0)	15 (5.0)
Lorcaserin	1 (0.3)	1 (0.3)
Liraglutide	0	0
Orlistat	3 (1.0)	0 (0.0)

Abbreviations: AOM: anti‐obesity medication; SMA: shared medical appointments.

### 3.2 Population description after propensity matching

Propensity matching identified 301 matched pairs that were well‐matched on all measured baseline variables (Table 3). The median age of the matched patients was 52 years, and 83% of patients were female. In this propensity matched population, 150 (49.8%) patients (in the SMA group) and 37 (12.3%) patients (in the non‐SMA group) were prescribed an AOM. Patients in the matched SMA group had a mean (SD) of 6.18 (3.31) endocrinology visits per patient as compared to a mean of 2.13 (2.07) visits per patient in the non‐SMA group.

**Table 3:** Baseline demographics after propensity matching

Variable Median (IQR) or n (%)	SMA N=301	Non‐SMA N=301	*P*‐value
Age (years)	51.97 (42.15, 60.02)	51.99 (40.15, 58.72)	.49
Gender			.83
Female	251 (83.4%)	249 (82.7%)	
Male	50 (16.6%)	52 (17.3%)	
Race			.77
White	157 (52.2%)	155 (51.5%)	
Black	132 (43.9%)	137 (45.5%)	
Other	12 (4%)	9 (3%)	
Marital status			.74
Married	139 (46.2%)	135 (44.9%)	
Other	162 (53.8%)	166 (55.1%)	
Insurance			.84
EHP	57 (18.9%)	59 (19.6%)	
Medicaid	52 (17.3%)	57 (18.9%)	
Medicare	75 (24.9%)	63 (20.9%)	
Other	4 (1.3%)	4 (1.3%)	
Private health insurance	113 (37.5%)	118 (39.2%)	
Median income (US Dollars)	50,866 (32,357, 65,043)	48,459 (37,142, 63,951)	.70
BMI (kg m^−2^)	40.59 (35.82, 45.95)	41.34 (34.96, 47.91)	.74
Weight (kg)	111.58 (95.71, 131.41)	112.63 (98.88, 133.49)	.63
Blood pressure (mmHg)			
Systolic	130 (119, 140)	130 (118.5, 139)	.60
Diastolic	78 (69, 85)	78 (71, 84)	.59
Comorbidities			
T2D	116 (38.5%)	126 (41.9%)	.41
Hypertension	195 (64.8%)	191 (63.5%)	.73
Dyslipidemia	182 (60.5%)	171 (56.8%)	.36

*Note.* Continuous variables summarized as median, IQR (interquartile range), categorical variables summarized as N (%).

Abbreviations: BMI: body mass index; EHP: Cleveland Clinic Employee Health Plan; IQR: interquartile range; SMA: shared medical appointments; T2D: type 2 diabetes mellitus.

### 3.3 Weight loss outcomes after propensity matching

**Figure 2:**
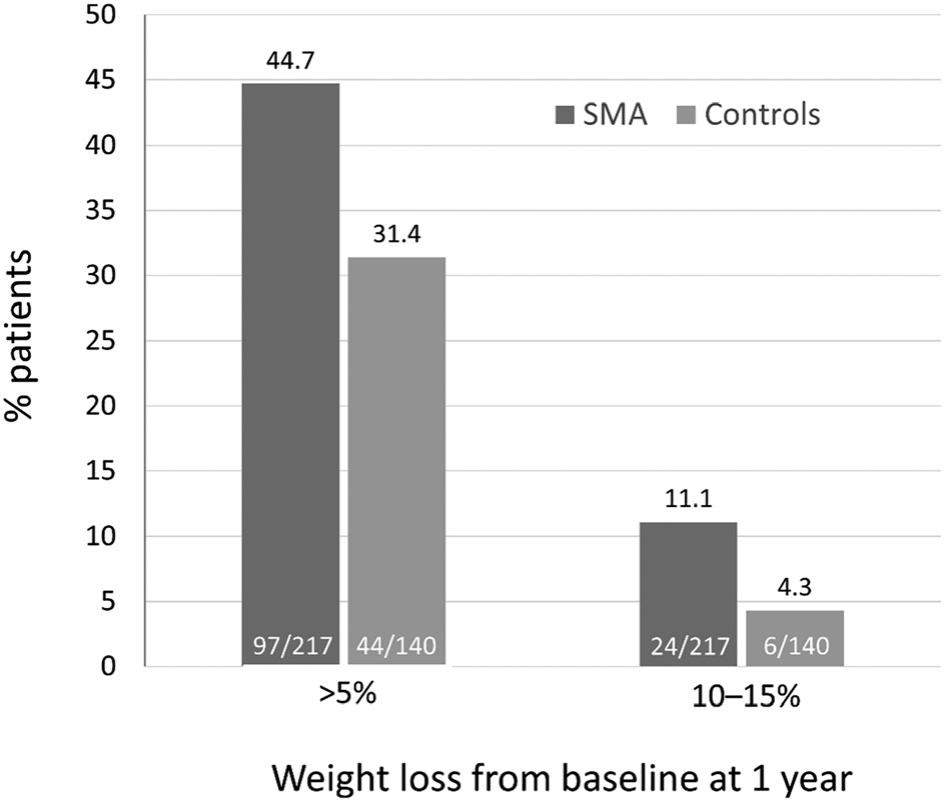
Categorical weight loss at 1 year (propensity‐matched Groups). SMA, shared medical appointments

Patients in the SMA group lost more weight at 6 months, 1 year and 2 years compared to the matched non‐SMA group (Table 4). The average weight loss at 1 year was 5.2% in the SMA group, compared to 1.8% in the non‐SMA group (*P*<.001). At 1 year, the proportions of patients who lost >5% and 10 to 15% of their baseline weight were higher in the SMA group as compared to the non‐SMA group (Figure 2).

**Table 4:** Weight loss outcomes after propensity matching

	SMA (N=301)		Non‐SMA (N=301)		*P*‐value
	N	Weight Loss (%) Mean (SD)	N	Weight Loss (%) Mean (SD)	
6 months	264	4.17 (5.79)	143	1.51 (5.91)	<.001
1 year	217	5.18 (7.05)	140	1.76 (7.54)	<.001
2 years	207	3.78 (7.95)	151	1.64 (9.06)	.02

Abbreviation: SMA: shared medical appointments.

## 4 DISCUSSION

In this longitudinal retrospective cohort study, patients enrolled in the SMA program had superior weight loss outcomes compared with a matched cohort of patients who had obesity addressed in individual medical appointments of usual care alone. In propensity‐matched comparisons, patients in the SMA group lost a mean of 5.2% of body weight at 1‐year, compared to patients in the non‐SMA group who experienced a mean 1.8% weight loss over the same period. These findings are relatively consistent with those reported in prior studies.^18^, ^19^, ^20^ A non‐controlled study of 216 participants of programmed SMAs for weight management reported an overall mean weight loss of 3.2%, and even higher (4.2‐4.3%) among patients who completed at least four of six SMA sessions (19). That study also reported that the programmed SMAs were estimated to be 4 times more cost effective and 7 times more effective than individual medical appointments for weight loss. In a study of 74 patients who participated in weight loss‐focused SMAs (mean baseline BMI, 35.3 kg m^−2^), this group lost an average of 1.0% of baseline weight compared to a non‐matched control group (mean baseline BMI, 29.4 kg m^−2^), which experienced an average increase of 0.8% of baseline weight.^18^ In a non‐controlled study of 222 patients who attended at least one SMA for weight loss over a 9‐month period, 41% of patients achieved a 5% weight loss.^20^

Prior evidence has shown that weight loss in the range of 3 to 5% of body weight can be associated with clinical health benefits such as improved glycaemic control^22^; the mean weight loss in the SMA group in our study was 5.2%. Furthermore, a 5% loss of body weight has been previously associated with improvement in several cardiovascular risk factors, including improvements in blood pressure and cholesterol^22^; this degree of weight loss was attained by almost half of patients in the SMA group. The degree of weight loss achieved by patients who attended SMAs is comparable to that of intensive lifestyle interventions outside of the medical setting, where participants have been reported to lose 3 to 8% of initial body weight.^23^, ^24^, ^25^, ^26^ Participants in SMAs may gain additional benefits from being seen by obesity‐focused medical providers, such as prescription of AOMs, identification of medications that promote weight gain and management of obesity‐related comorbidities. SMAs also provide opportunity for comprehensive medical care of obesity, such as addressing nutrition, exercise, sleep and stress management. Patients in SMAs can learn from their peers' experiences to gain a better understanding of their obesity treatment options.^5^

However, like longitudinal studies of weight loss, our data show that weight loss maintenance over 2 years was a challenge. In the SMA group, patients lost 5.2% of body weight at 1 year, but at 2 years, median weight loss was 3.8%. This may be due to inconsistent SMA attendance, patient dropout from SMAs or the use of phentermine, an AOM approved only for short‐term use. In other studies, only 20% of patients were able to maintain their weight loss 1 year after treatment end.^27^, ^28^ Weight loss maintenance is difficult due to the emergence of a hormone profile and physiologic adaptations that promote weight regain.^29^ These data highlight the need for chronic obesity management. In order to adequately treat patients, medical management needs to be continued long term, and there is a need for continual patient engagement in obesity treatment. Prior research has identified factors such as follow‐up visit frequency^30^, ^31^ and interdisciplinary intervention^32^ as contributing positively to weight loss over periods of 1 to 2 years, both of which are characteristics of the SMA approach.

All the patients in our study were eligible to take a Food and Drug Administration‐approved AOM due to their diagnosis of obesity. However, AOM prescriptions varied markedly between the SMA group (49.8% of patients prescribed an AOM) and the matched non‐SMA group (12.3% of patients prescribed an AOM), possibly due to differences in provider experience in prescribing AOMs Table 2. The majority of AOM prescriptions were phentermine hydrochloride, approved only for 3 months of use. Thus, isolating the contribution of AOMs vs the effect of SMAs on weight loss outcomes is difficult to discern in our study. However, in clinical trials, AOMs have been shown to produce an additional weight loss of 3 to 7% compared to lifestyle interventions alone.^33^ Thus, increased access to AOMs may be an intermediate benefit of SMAs regarding weight loss efficacy.

One limitation in this study is that due to its retrospective nature, patients in the SMA group differed from those in the non‐SMA group. Patients in the SMA group were more likely to be female, be covered under the Cleveland Clinic Employee Health Plan, have a higher baseline BMI and were less likely to be Caucasian. In an attempt to address these differences, propensity score matching was used to identify cohorts that were balanced in selected baseline variables. This allowed for control of potential confounding factors in a large population of patients who attended SMAs and individual medical appointments for obesity. However, limitations of propensity score matching apply to this study, particularly that unmeasured variables, such as motivation to participate in a structured group program for weight management or patient interest in AOMs, may have introduced confounding. The SMA cohort was likely to have been comprised largely of motivated individuals actively seeking weight loss treatment, while those counselled through individual appointments may have been more passive in their approach to weight loss.

Another limitation is that although individual medical appointments were conducted in the Department of Endocrinology, level of care was left to the discretion of the individual provider, likely reflecting the heterogeneity of obesity treatment provided in real‐world clinics. Given the challenges of intensive obesity treatment, it is possible that some patients in the non‐SMA group may have received minimal obesity care. The non‐SMA patients also had fewer mean numbers of endocrinology visits compared to the SMA cohort. Further, patients with missing weight values at specific timepoints, most likely due to patients not following‐up in clinic, were excluded from that analysis. Excluding these patients may have added selection bias to our results.

In addition, the use of coding to identify patients with obesity in the non‐SMA group has limitations; in that, it cannot be confirmed with absolute certainty that robust discussions around weight management occurred between providers and these patients. However, given evidence that in the United States, only 28% of patients with a BMI≥30 kg m^−2^ are coded for obesity,^34^ it is clear that this is not a common practice and thus, is likely to indicate, that some level of focused discussion about weight management did occur at that visit.

Finally, it is possible that referral to any type of weight management counselling outside of a patient's primary care provider leads to better weight loss, whether as a group or individual. For example, data from the POWER‐UP trial^35^ found that patients who participated in regular weight counselling activities by a medical assistant in addition to ongoing visits with their primary care physician (PCP) lost more weight than did those who received only counselling from the PCP. The group strategy may still have cost effectiveness benefits compared to individual appointments and be preferred by some, but not all, patients.

With the increasing time constraints placed on physicians, SMAs allow providers to increase their productivity by seeing more patients in a given timeframe, thus allowing for a more appropriate amount of time to specifically address obesity management. Further, patients have more time with the provider(s) leading the SMAs. Coordinated SMA programs may also allow patients to access more tools that can aid in chronic weight management, evidenced by the higher percentage of AOM utilization in the SMA group as compared to the non‐SMA group. Our findings suggest that SMAs in this context might offer a means of achieving clinically meaningful weight loss in some patients. This concept should be considered and researched further as a way to provide effective treatment to a higher number of patients with obesity. Future work should further evaluate differences in weight loss outcomes between patients participating in SMAs and taking AOMs compared to patients participating in SMAs without use of AOMs.

## FUNDING

This analysis was supported by funding provided by Novo Nordisk (Plainsboro, NJ). The authors acknowledge the assistance of Sandra Westra, PharmD of Churchill Communications (Maplewood, New Jersey), with preparing this manuscript for submission, which was funded by Novo Nordisk.

## DATA AVAILABILITY STATEMENT

Restrictions apply to the availability of data generated or analyzed during this study to preserve patient confidentiality or because the data were used under license. The corresponding author will on request detail the restrictions and any conditions under which access to some data may be provided.

## CONFLICTS OF INTEREST STATEMENT

XJ reports receiving research funding from Merck and Novo Nordisk, Inc. within the past 12 months. AM reports receiving research funding from Merck, Boehringer Ingelheim, Novartis and Novo Nordisk, Inc. within the past 12 months. WW, RG, TMH and AR report being employees of Novo Nordisk, Inc., and holding company stock. JMB and MWK report receiving research funding from Merck and Novo Nordisk, Inc. within the past 12 months. ADM has received research support from Merck, Novo Nordisk, Inc. and the Agency for Healthcare Research and Quality K08 HS024128 within the past 12 months. KMP reports receiving research funding from Novo Nordisk, Inc. and Merck; receiving consulting fees from Novo Nordisk, Inc. Sanofi, Eli Lilly, Bayer and Merck; and participating in the speaker bureaus of Novo Nordisk, Inc., Merck, AstraZeneca within the past 12 months. BB has received consulting fees and has ongoing research support from Novo Nordisk. KS and ERP have no conflicts of interest to declare.

## References

[1] American Medical Association . H440.842 Recognition of Obesity as a Disease. 2013. Available at: Https://Www.Ama‐Assn.Org/Ssl3/Ecomm/PolicyFinderForm.Pl?Site=www.Ama‐Assn.Org&uri=/Resources/Html/PolicyFinder/Policyfiles/HnE/H‐440.842.HTM.

[2] Simon R , Lahiri SW . Provider practice habits and barriers to care in obesity management in a large multicenter health system. Endocr Pract. 2018;24:321‐328. 29561192 10.4158/EP-2017-0221

[3] Xia Y , Kelton CM , Guo JJ , Bian B , Heaton PC . Treatment of obesity: pharmacotherapy trends in the United States from 1999 to 2010. Obesity (Silver Spring). 2015;23:1721‐1728. 26193062 10.1002/oby.21136

[4] Moyer V . Screening for and management of obesity in adults: U.S. Preventive Services Task Force recommendation statement. Ann Intern Med. 2012;157:373‐378. 22733087 10.7326/0003-4819-157-5-201209040-00475

[5] Shibuya K , Pantalone KM , Burguera B . Obesity: Are shared medical appointments part of the answer? Cleve Clin J Med. 2018;85:699‐706. 30192733 10.3949/ccjm.85a.18006

[6] Ramdas K , Darzi A . Adopting innovations in care delivery—the case of shared medical appointments. N Engl J Med. 2017;376:1105‐1107. 28328325 10.1056/NEJMp1612803

[7] Kirsh SR , Aron DC , Johnson KD , et al. A realist review of shared medical appointments: How, for whom, and under what circumstances do they work? BMC Health Serv Res. 2017;17:1‐13. 28160771 10.1186/s12913-017-2064-z PMC5291948

[8] Ramdas K , Tesiberg E , Tucker AL . Four ways to reinvent service delivery. Harvard Business Review. December 2012. Available at: https://hbr.org/2012/12/four-ways-to-reinvent-service-delivery. .

[9] Trento M , Passera P , Tomalino M , et al. Group visits improve metabolic control in type 2 diabetes ‐ a 2‐year follow up. Diabetes Care. 2001;24:995‐1000. 11375359 10.2337/diacare.24.6.995

[10] Trento M , Passera P , Bajardi M , et al. Lifestyle intervention by group care prevents deterioration of type II diabetes: a 4‐year randomized controlled clinical trial. Diabetologia. 2002;45:1231‐1239. 12242455 10.1007/s00125-002-0904-8

[11] Trento M , Passera P , Borgo E , et al. A 5‐year randomized controlled study of learning, problem solving ability, and quality of life modifications in people with type 2 diabetes managed by group care. Diabetes Care. 2004;27:670‐675. 14988283 10.2337/diacare.27.3.670

[12] Trento M , Passera P , Borgo E , et al. A 3‐year prospective randomized controlled clinical trial of group care in type 1 diabetes. Nutr Metab Cardiovasc Dis NMCD. 2005;15:293‐301. 16054554 10.1016/j.numecd.2004.12.005

[13] Trento M , Gamba S , Gentile L , et al. Rethink organization to iMprove education and outcomes (ROMEO): a multicenter randomized trial of lifestyle intervention by group care to manage type 2 diabetes. Diabetes Care. 2010;33:745‐747. 20103547 10.2337/dc09-2024 PMC2845019

[14] Naik AD , Palmer N , Petersen NJ , et al. Comparative effectiveness of goal setting in diabetes mellitus group clinics: randomized clinical trial. Arch Intern Med. 2011;171:453‐459. 21403042 10.1001/archinternmed.2011.70 PMC3132209

[15] Raballo M , Trevisan M , Trinetta AF , et al. A study of patients' perceptions of diabetes care delivery and diabetes: propositional analysis in people with type 1 and 2 diabetes managed by group or usual care. Diabetes Care. 2012;35:242‐247. 22210565 10.2337/dc11-1495 PMC3263876

[16] Menon K , Mousa A , de Courten PJ , et al. Shared medical appointments may be effective for improving clinical and behavioral outcomes in type 2 diabetes: a narrative review. Front Endocrinol. 2017;8:1‐12. 10.3389/fendo.2017.00263 PMC5632846 29046662

[17] Wadsworth KH , Archibald TG , Payne AE , et al. Shared medical appointments and patient‐centered experience: a mixed‐methods systematic review. BMC Family Practice. 2019;20:1‐13. 31286876 10.1186/s12875-019-0972-1 PMC6615093

[18] Palaniappan LP , Muzaffar AL , Wang EJ , Wong EC , Orchard TJ , Mbbch M . Shared medical appointments: promoting weight loss in a clinical setting. J Am Board Fam Med. 2011;24:326‐328. 21551406 10.3122/jabfm.2011.03.100220 PMC3217311

[19] Egger G , Stevens J , Volker N , Egger S . Programmed shared medical appointments for weight management in primary care: an exploratory study in translational research. Aust J Gen Pract. 2019;48:681‐688. 31569313 10.31128/AJGP-05-19-4940

[20] Yager S , Parker M , Luxenburg J , et al. Evaluation of multidisciplinary weight loss shared medical appointments. J Am Pharm Assoc 2013; 2020 Jan‐Feb;60(1): 93‐9930358. 10.1016/j.japh.2019.07.014 31466900

[21] (ACS) ACS . US Census Bureau.

[22] Wing RR , Lang W , Wadden TA , et al. Look AHEAD Research group. Benefits of modest weight loss in improving cardiovascular risk factors in overweight and obese individuals with type 2 diabetes. Diabetes Care. 2011;34:1481‐1486. 21593294 10.2337/dc10-2415 PMC3120182

[23] Knowler WC , Barrett‐Connor E , Fowler SE , et al. Diabetes Prevention Program Research Group. Reduction in the incidence of type 2 diabetes with lifestyle intervention or metformin. N Engl J Med. 2002;346:393‐403. 11832527 10.1056/NEJMoa012512 PMC1370926

[24] Eriksson J , Lindström J , Valle T , et al. Prevention of Type II diabetes in subjects with impaired glucose tolerance: The Diabetes Prevention Study (DPS) in Finland. Study design and 1‐year interim report on the feasibility of the lifestyle intervention programme. Diabetologia. 1999;42:793‐801. 10440120 10.1007/s001250051229

[25] Look AHEAD Research Group , Pi‐Sunyer X , Blackburn G , et al. Reduction in weight and cardiovascular disease risk factors in individuals with one‐year results of the look AHEAD trial. Diabetes Care. 2007;30:1374‐1383. 17363746 10.2337/dc07-0048 PMC2665929

[26] Burguera B , Jesús Tur J , Escudero AJ , et al. An intensive lifestyle intervention Is an effective treatment of morbid obesity: the TRAMOMTANA study‐a two‐year randomized controlled clinical trial. Int J Endocrinol. 2015;2015:1‐12. 10.1155/2015/194696 PMC4518148 26257780

[27] Wing RR , Hill JO . Successful weight loss maintenance. Annu Rev Nutr. 2001;21:323‐341. 11375440 10.1146/annurev.nutr.21.1.323

[28] Middleton KM , Patidar SM , Perri MG . The impact of extended care on the long‐term maintenance of weight loss: A systematic review and meta‐analysis. Obes Rev. 2012;13:509‐517. 22212682 10.1111/j.1467-789X.2011.00972.x

[29] Sumithran P , Proietto J . The defence of body weight: a physiological basis for weight regain after weight loss. Clin Sci (Lond). 2013;124:231‐241. 23126426 10.1042/CS20120223

[30] Compher CW , Hanlon A , Kang Y , Elkin L , Williams NN . Attendance at clinical visits predicts weight loss after gastric bypass surgery. Obes Surg. 2012;22:927‐934. 22161257 10.1007/s11695-011-0577-9

[31] Bachar A , Hermoni D , Livshits G , et al. Late successful weight reduction and maintenance among overweight and obese adults—a two‐year retrospective study. Diab Res Clin Pract. 2014;106:511‐521. 10.1016/j.diabres.2014.09.055 25458338

[32] Tapsell LC , Lonergan M , Batterham MJ , et al. Effect of interdisciplinary care on weight loss: a randomised controlled trial. BMJ Open. 2017;7:e014533 1‐11 10.1136/bmjopen-2016-014533 PMC5734361 28710205

[33] Yanovski SZ , Yanovski JA . Long‐term drug treatment for obesity: a systematic and clinical review. JAMA. 2014;311:74‐86. 24231879 10.1001/jama.2013.281361 PMC3928674

[34] Mocarski M , Tian Y , Smolarz BG , McAna J , Crawford A . Use of International Classification of Diseases, Ninth Revision Codes for Obesity: Trends in the United States from an electronic health record‐derived database. Popul Health Manag. 2018;21:222‐230. 28949834 10.1089/pop.2017.0092 PMC5984561

[35] Wadden TA , Volger S , Sarwer DB , et al. A two‐year randomized trial of obesity treatment in primary care practice. N Engl J Med. 2011;365:1969‐1979. 22082239 10.1056/NEJMoa1109220 PMC3282598

